# Electroencephalography in Assessment of Autism Spectrum Disorders: A Review

**DOI:** 10.3389/fpsyt.2021.686021

**Published:** 2021-09-29

**Authors:** Maja Milovanovic, Roberto Grujicic

**Affiliations:** ^1^Department for Epilepsy and Clinical Neurophysiology, Institute of Mental Health, Belgrade, Serbia; ^2^Faculty for Special Education and Rehabilitation, University of Belgrade, Belgrade, Serbia; ^3^Clinical Department for Children and Adolescents, Institute of Mental Health, Belgrade, Serbia

**Keywords:** autistic spectrum disorder, electroencephalography, epilepsy, epileptiform discharges, excitation/inhibition imbalance

## Abstract

Electroencephalography (EEG) can further out our understanding of autistic spectrum disorders (ASD) neurophysiology. Epilepsy and ASD comorbidity range between 5 and 46%, but its temporal relationship, causal mechanisms and interplay with intellectual disability are still unknown. Epileptiform discharges with or without seizures go as high as 60%, and associate with epileptic encephalopathies, conceptual term suggesting that epileptic activity can lead to cognitive and behavioral impairment beyond the underlying pathology. Seizures and ASD may be the result of similar mechanisms, such as abnormalities in GABAergic fibers or GABA receptor function. Epilepsy and ASD are caused by a number of genetic disorders and variations that induce such dysregulation. Similarly, initial epilepsy may influence synaptic plasticity and cortical connection, predisposing a growing brain to cognitive delays and behavioral abnormalities. The quantitative EEG techniques could be a useful tool in detecting and possibly measuring dysfunctions in specific brain regions and neuronal regulation in ASD. Power spectra analysis reveals a U-shaped pattern of power abnormalities, with excess power in the low and high frequency bands. These might be the consequence of a complicated network of neurochemical changes affecting the inhibitory GABAergic interneurons and their regulation of excitatory activity in pyramidal cells. EEG coherence studies of functional connectivity found general local over-connectivity and long-range under-connectivity between different brain areas. GABAergic interneuron growth and connections are presumably impaired in the prefrontal and temporal cortices in ASD, which is important for excitatory/inhibitory balance. Recent advances in quantitative EEG data analysis and well-known epilepsy ASD co-morbidity consistently indicate a role of aberrant GABAergic transmission that has consequences on neuronal organization and connectivity especially in the frontal cortex.

## Introduction

Autism spectrum disorders (ASD) are manifested by persistent impairments in social communication and interaction, in addition with restricted, repetitive patterns of behavior, interests, and activities (1). The co-morbidity of ASD and epilepsy is now well-documented (2). The prevalence of epilepsy in patients with ASD is substantially greater than in the general population (1.8-60%; 0.5-0.7%, respectively) (3, 4). Furthermore, in patients with autism, the incidence of epileptiform activity (EA) on electroencephalography (EEG) ranges from 23.6 to 60.8% (5–8).

EEG has been the primary method used to record and describe epileptiform paroxysmal activity, which occur more often in ASD. EEG recordings may also be used to examine functional connectivity across various brain areas over time through EEG coherence, which is a quantitative assessment of the connection between two EEG signals' frequency spectra (9). This useful feature can help us better understand impaired interconnections across brain areas that have been revealed by functional MRI studies in patients with ASD (10–16).

The aim of this review was to provide the summary of recent literature on the EEG findings in ASD that contribute to the understanding the neurophysiology of ASD. First, we present available data on the neurobiological mechanisms underlying ASD based on epilepsy and EA comorbidity, and then based on quantitative EEG assessments.

## Bidirectional Relation Between ASD and Epilepsy/Epileptiform Abnormalities

According to a meta-analysis of 23 studies, the prevalence of epilepsy in patients with ASD and intellectual impairment is 21.5% (2,150/10,000) and 8% (800/10,000) in patients with ASD without intellectual disability (17). This is especially important due to the fact that 31% of children with ASD have an intellectual impairment [intelligence quotient (IQ) < 70], and 25% have IQ scores in the borderline range (IQ 71–85) (18). In a sample of 5,185 children with ASD, Viscidi et al. (19) demonstrated a significant connection between seizures and cognitive impairment. They demonstrated an inverse connection between IQ and epilepsy, i.e., for every 1 standard deviation increase in IQ, the probability of developing epilepsy reduced by 47% in children over the age of 10.

ASD patients have high rate of interictal epileptic discharges (IEDs) even in the absence of definite clinical seizures—subclinical epileptic discharges (SEDs). In our previous study of patients with severe ASD of unexplained cause (*n* = 112), prevalence of epilepsy was 15.2%. IEDs in awake EEG recordings were found in 20.4%, and in sleep EEG recordings in 41.3% of cases (20). SEDs and the diagnosis of epilepsy occurred more frequently in the group of non-verbal ASD patients (15.8%; 21.0%, respectively) compared to verbal ones (7.5%; 11.3%, respectively), but the difference was not statistically significant. Patients with ASD without epilepsy and/or SEDs showed slight tendency to have better motor skills scores on Vineland adaptive behavior scale II, compared to the group with epilepsy and/or SEDs. Other symptoms of ASD didn't differ significantly between the groups. Similarly, according to Turk et al., children with ASD and epilepsy were more likely to have intellectual impairment, motor problems, developmental delays, and demanding behaviors than children with only ASD (21).

Specifically, this comorbidity is reciprocal; according to review of 19 studies, the median overall period prevalence of ASD in individuals with epilepsy was 9.0% (4).

ASD associated with seizures, language regression, or motor impairments might represent clinical subtypes, and could aid genetic research into the etiology of ASD (22). A number of the genes linked to ASD have also been linked to epilepsy. Synaptic transmission and DNA methylation/chromatin remodeling are the major functions of these genes (23).

Both early-onset epilepsy and ASD symptomatology characterize genetic disorders such as Fragile X (FXS), tuberous sclerosis, Rett syndrome, maternal duplications on chromosome 15q11.2-q13.1 (Dup15q), and Phelan-McDermid syndromes (24). Comorbidity with intellectual impairment is also a feature of these hereditary disorders (25). Dup15q syndrome is caused by overexpression of several genes, including ubiquitin ligase E3A (UBE3A) and a cluster of GABAA receptor subunits (26). Single-nucleotide polymorphisms in GABA receptor subunit genes have been linked to ASD and epilepsy in association studies (27, 28).

Additionally, number of existing evidence point to the complex association between ASD and epileptic encephalopathies (EE). EE are a group of neurological disorders that occur in early life and present with characteristic symptoms: different types of seizures and/or EA in EEG, as well as severe cognitive, behavioral, and neurological deficits (Table 1). According to ILAE definition: “EE is a conceptual term suggesting that EA, seizures, or IEDs can lead to cognitive and behavioral impairment, including ASD, above and beyond what might be expected from the underlying pathology” (29). This definition is based on the finding of various mechanisms through which EA affects brain development, such as disruption of anatomical and functional characteristics of the brain at various stages of development (30). Very important question that comes from EE is whether the seizures and IEDs independently worsen the development of ASD, especially if they emerged at the first 2 years, during the period of rapid brain growth and maturation (31, 32).

**Table 1:** Types of epileptic encephalopathies.

**Epileptic encephalopathies**
Early-onset epileptic encephalopathy
Early infantile epileptic encephalopathy (Otahara syndrome)
Early myoclonic encephalopathy
Epilepsy of infancy with migrating focal seizures
Infantile spasms (IS),
Myoclonic infantile epilepsy (Dravet syndrome) (DS)
Epilepsy with myoclonic-astatic seizures (Doose syndrome)
Lennox-Gastatut syndrome (LGS)
Epilepsy aphasia spectrum:
Landau-Kleffner syndrome (LKS)
Continuous spike-wave discharges in slow wave sleep syndrome (CSWSS)

Core ASD features in monogenic EE are repetitive behaviors with a lower order cognitive component (e.g., motor stereotypies) and behaviors with higher cognitive implications (e.g., perseverations and obsessions) (33).

The genetic overlap between ASD and EE might potentially be seen as a connection between the two disorders. According to McTague et al., 62 genes could be connected with the development of EE (34). Out of these, 34 genes are proven to be the significant risk factors for development of ASD. Also, certain types of EE occur more frequently with ASD symptoms compared to others. Especially, EEs associated with mutations in CDKL5 (encoding cyclin-dependent kinase-like 5), SCN1A (encoding sodium voltage-gated channel alpha subunit 1), and SLC6A1 (encoding GABA transporter 1) have a high co-occurrence of ASD features (35–37).

Tuberous sclerosis complex (TSC) is a disorder of mTOR signaling caused by mutations in TSC1 and TSC2, associated with epilepsy (up to 80%), especially infantile spasms (IS) (38–40) and high prevalence rates of ASD (up to 50%) (41, 42). Not all patients with IS develop ASD, and not all individuals with ASD had prior IS, implying that ASD and IS may be two distinct end-results of a shared CNS abnormality in the TSC population (43).

Dravet syndrome (DS) is another example of a condition in which cognitive outcomes do not always correspond to seizure intensity. Syndrome is characterized by myoclonic seizures in infancy, often associated with fever, which progresses to other seizure types (44). DS is frequently caused by mutations in the SCN1A (45), that have been linked to autism, also (46).

In epilepsy-aphasia spectrum, amount of EA are responsible for developmental regression (47). Landau-Kleffner syndrome (LKS) is characterized by language regression and temporal lobe epileptiform discharges (47). It has been linked to mutations in the GRIN2A gene (48), which codes for the GluN2A protein present in speech and language cortical areas. Autistic symptoms may be present in a clinical picture of LKS, in addition to cognitive delay and EE (49).

**Figure 1:**
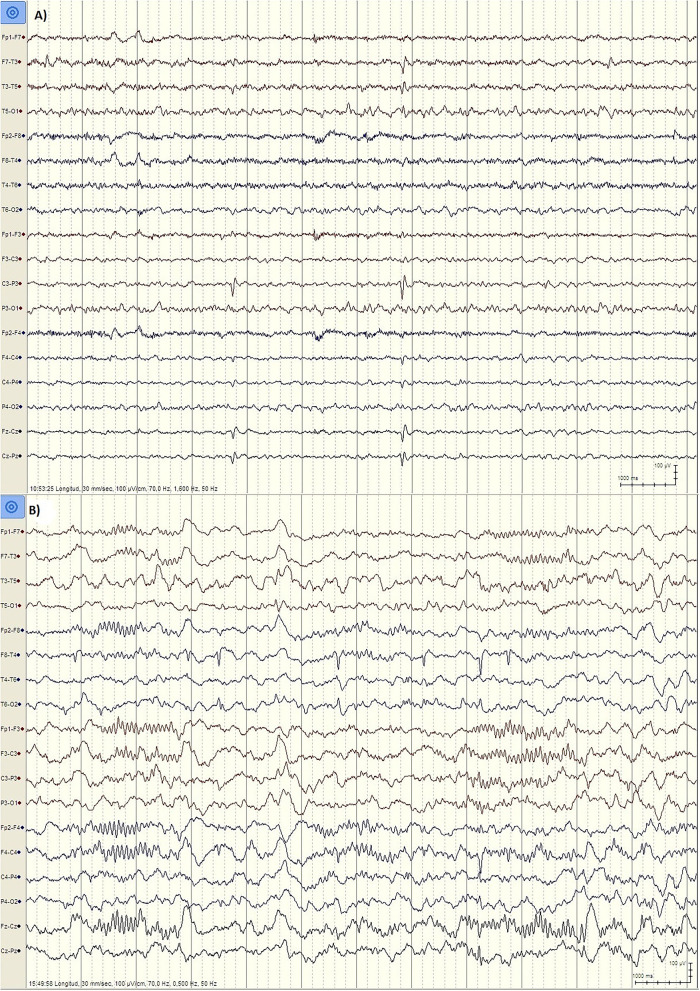
EEG findings in ASD patients. **(A)** Patient PR, male, 6 years. EEG during wakefulness (Bipolar longitudinal montage, Sensitivity 100 μV/cm, High pass filter 1.6 Hz, Low pass filter 70 Hz): Left centroparietal spikes (P3, C3). **(B)** Patient JL, male, 5 years old. EEG during N2 stage Sleep (Bipolar longitudinal montage, Sensitivity 100 μV/cm, High pass filter 0.5 Hz, Low pass filter 70 Hz): Right temporal spikes (T4, T6).

**Figure 2:**
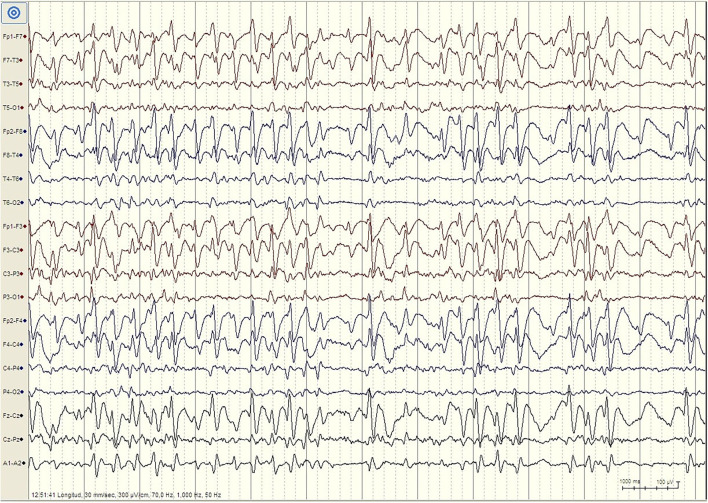
EEG finding in patient with Landau-Kleffner Syndrome. Patient LM, female 6 years. EEG during N2 Stage Sleep (Bipolar Longitudinal montage, Sensitivity 300 μV/cm, High pass filter 1 Hz, Low pass filter 70 Hz): High amplitude generalized spike–waves with variable frequency, mostly <3Hz. There is repeated fragmentation of these discharges, which still occupy more than 80% of the epoch. For the whole sleep period, the spike–waves occupied more than 85% of slow waves sleep, constituting continuous spike-waves during slow sleep (CSWS).

But there are important differences between developmental regression in LKS and ASD. In LKS, after relatively typical early development, regression primarily affects language between 3 and 9 years of age, but behavioral problems are considerably less common and may be caused by speech impairment or cognitive decline (49). In ASD, language and social skills loss typically begins before the age of three, and the regression might be clinically mild (e.g., loss of single words, reduced gesturing) (50). The epileptic discharges associated with developmental regression in ASD includes focal spikes that can be infrequent (51) (Figure 1). Language loss in LKS is severe, with loss of completely developed language, and EEG is characterized by frequent temporoparietal spikes, which are notably activated by slow wave sleep (SWS), or with the EEG pattern of continuous spike-wave in sleep (CSWS) (Figure 2). CSWS is defined as almost continuous 1.5-2 Hz spike-waves, that takes up >85% of SWS (29). However, even a spike-wave index in sleep (SWI) of more than 50% affect child's development, including the development of autistic symptoms as well as cognitive, behavioral, and/or motor regression (52).

The clinical assessment of patients with developmental regression, epilepsy and ASD has to be comprehensive and needs to include multiple variables. Some of the important variables include the type of regression (language vs. autistic), the age of onset of seizures or IEDs, the detailed location and description, as well as assessing the amount and persistence of EA. These variables can significantly influence further clinical management and treatment plan (25). There is still no existing evidence that strongly supports treatment with antiepileptic drugs in ASD, if the EEG demonstrates infrequent spikes, in absence of seizures. However, there's still an ongoing debate among experts concerning the use of pharmacotherapy in these patients. It is important to note that there are several clinical studies which demonstrated improvement in ASD symptomatology when treated with anticonvulsants (53, 54), but further studies are needed to confirm these findings.

Children with EE are more prone to develop ASD; hence, in addition to urgent pharmacological treatment with protocols for EE, early behavioral, communication, and educational interventions should be addressed as part of their comprehensive management (25).

## Quantitative EEG Findings in ASD

The quantitative EEG techniques could be a useful tool in detecting and possibly measuring dysfunctions in specific brain regions and neuronal regulation in ASD. Scientific interest in identifying EEG biomarkers of ASD, with a focus on spectral power, coherence, and hemisphere asymmetry, recently raised.

Wang and colleagues, in their review of resting-state EEG studies in ASD, reported a potential “U-shaped" profile of EEG power spectra in ASD as compared to typically developing controls, with excess power in theta and gamma frequency bands and decreased power in alpha frequency band (55). Other research has found alpha band asymmetry (56) between hemispheres in infants at risk for ASD.

EEG coherence, a key approach of EEG functional connectivity research, has been used to investigate how brain regions communicate in real time. When two signals of the same frequency are active and have a constant phase relationship across time, they are termed coherent, meaning there is a high degree of coordinated activity between the brain areas creating them (57–59).

Two EEG coherence investigations in ASD patients found under and over-connectivity in distinct frequency bands (60, 61). Increased local coherence has been found across the frontal area in delta band (62), as well as over the left frontal and temporal regions in the theta band (61). In contrast, reduced intrahemispheric and interhemispheric local coherence in all brain regions has been reported in delta and theta bands (63), and, also, reduced local coherence over the frontal region in delta (62) and alpha bands (63). Machado et al. discovered that ASD children had considerably higher intrahemispheric long-range coherence in the left hemisphere, confirming the notion of over functional connectivity in ASD (64). Carson et al., on the other hand, found that children with ASD had reduced long-distance coherence at the alpha frequency in resting state (65). Children with ASD have decreased interhemispherical and intrahemispherical coherence in the delta and theta frequency bands, according to Coben et al. (63). Wang et al. showed higher coherence of short and long-distance connections in children with ASD compared to controls, which was related to clinical severity scale scores (66).

According to the theory of nerve pruning, throughout normal brain development, neurons matures by the myelination, and is further pruned and modified (67, 68). Pruning and synaptogenesis allow continuous changes in both short- and long-range neuronal circuitry in the normal brain growth, leading to a weakening of the functional connections between the neighboring areas of the brain, and simultaneously strengthening connections between distant brain regions (69). This mechanism may be disrupted in developmental diseases such as autism, resulting in aberrant brain connections. There is evidence that synaptic disruption occurs in ASD at both the local level of single axons and the broader level of brain networks (70, 71). Using EEG coherence to examine electrical connection patterns, researchers may be able to analyze the resulting differences in brain function between persons with and without ASD (72, 73).

## Neurobiological Mechanisms Underlying ASD Based on EEG Findings: Excitation/Inhibition Imbalance

The co-morbidity of ASD and epilepsy could be currently explained by the most widely accepted theory of brain hyperexcitability. Various structural and functional defects of genetic, metabolic, immune or environmental etiologies, could permanently compromise balance in excitation (E) and inhibition (I) circuits (74).

The interaction of ASD, epilepsy, and intellectual disability led to the hypothesis that ASD and epilepsy are outcomes of similar processes, such as dysregulation of E/I balance, caused by defects in GABAergic fibers, particularly GABAergic interneurons maturation, or GABA receptor function (75). Several genes involved in the function of ion channels that play key functions in the brain, such as SCN1A or GABAA receptors, have been implicated in ASD and EEs (25). The increased activity of glutamate receptor signaling can also lead to hyperexcitability (76). Primary epilepsy, on the other hand, may affect synaptic plasticity and cortical connection, predisposing a developing brain to cognitive delays and behavioral abnormalities (25, 77, 78).

Based on quantitative EEG findings, U-shaped power spectra profile may be attributed to abnormal functioning of GABAergic tone in inhibitory circuitry, which influences the functional and developmental plasticity of the brain and decrease power of high-frequency and low-frequency bands while increasing the power of middle-range frequencies (79). This profile could be caused by affected GABAergic interneurons that has modulating role on excitatory pyramidal cells (55). The gamma band activity has been related to dendritic GABAergic inhibitory dysfunction (80). GABAergic interneurons synapsis with Nmethyl-D-aspartate receptors (NMDAR) on glutamatergic neurons causes thalamocortical delta oscillations, which are regulated by dopaminergic neurons in the thalamus (81).

Functional connectivity studies using EEG coherence revealed overall local over-connectivity and long-range under-connectivity, as well as increased power of delta frequency in the frontal brain region in individuals with ASD (66, 82, 83). Those findings consistently point to a role of aberrant GABAergic transmission on neuronal organization and connectivity especially in the frontal cortex.

There is evidence that GABAergic interneuron growth and connections in the prefrontal and temporal cortices are altered in ASD (84), which could lead to E/I imbalance (85). In post-mortem brain samples of ASD cases, it was found that neocortical minicolumns, elemental modular microcircuits made up of excitatory pyramidal neurons surrounded by GABAergic inhibitory neurons, were reduced, which could results in inhibitory circuits disruption (84, 86).

GABAergic disorders can affect early development, because prenatally GABA has the role of excitatory trophic factor, leading to the growth and binding of dendrites (87). In the mature brain, GABA acts as an inhibitory transmitter. Defects in GABAergic signaling, especially shifting the E/I balance toward excitatory transmission, may thereby explain some of the characteristics of ASD (88).

## Discussion

Although there is a number of etiological hypotheses, one of the most researched etiological mechanisms in the development of ASD in the last decade is the E/I imbalance in key cortical and subcortical neuronal circuits (75, 89, 90). This hypothesis was first proposed in the seminal work of Rubenstein and Merzenich in 2003 (74). To date, there is an abundance of evidence that support this model both from preclinical (90) and in clinical (89) perspectives.

When processed and analyzed with the most advanced techniques, the EEG might be a valuable approach in clinical and scientific studies of ASD neurophysiological substrates. Nevertheless, the quantitative EEG techniques could be a useful tool in detecting and possibly measuring dysfunctions in specific brain regions and neuronal regulation in ASD. Advances in quantitative EEG analysis in recent years and well-known epilepsy ASD co-morbidity consistently indicate a role of aberrant GABAergic transmission that has consequences on neuronal organization and connectivity especially in the frontal cortex.

Bosl and colleagues in 2011 provided initial data that highlighted the role of brain connectivity in early development (91). The authors investigated EEG complexity in newborns at risk for ASD compared to normal controls, finding that the infants-at-risk has less brain complexity. This study shows that decreased connectivity during early development is linked to the likelihood of ASD, although no definitive diagnostic outcome for infants was obtained in this investigation. As a result, it is uncertain if connectivity has any predictive value for autism risk.

Although, at this point, EEG research in ASD shows promising results in early detection and prediction of atypical brain development (92), EEG is still not a reliable clinical diagnostic tool for ASD due to its low sensitivity or specificity. However, the data presented in this review strongly suggest that EEG should be a complementary technique to the existing methods in diagnostic process.

Also, the available data on this matter indicate that further research is needed to provide better understanding of different electrophysiological features of high importance which could fill in major gaps in understanding pathophysiology and assessment of ASD. Due to the fact that ASD is a neurodevelopmental disorder, primary research focus should be on longitudinal studies which could potentially strengthen the available findings and also define the developmental stages of ASD. We strongly believe that combining the new approaches in EEG methodology with already established ones, could potentially open a new perspective on ASD assessment and eventually lead to new early diagnosis, early intervention and prevention strategies.

## Author Contributions

MM contributed with literature research, analysis, expertise, and interpretation, as well as the writing and reviewing process. RG contributed with the research, interpretation, and writing of the paper, as well as reviewing and technical work. All authors contributed to the article and approved the submitted version.

## Conflict of Interest

The authors declare that the research was conducted in the absence of any commercial or financial relationships that could be construed as a potential conflict of interest.

## Publisher's Note

All claims expressed in this article are solely those of the authors and do not necessarily represent those of their affiliated organizations, or those of the publisher, the editors and the reviewers. Any product that may be evaluated in this article, or claim that may be made by its manufacturer, is not guaranteed or endorsed by the publisher.
